# Supplementary material for: Whole Genome Resequencing of 205 Avocado Trees Unveils the Genomic Patterns of Racial Divergence in the Americas
Source: Int J Mol Sci. 2025 Oct 24;26(21):10353. doi: 10.3390/ijms262110353 (PMC12610134; doi:10.3390/ijms262110353)
Supplement: Supplementary file 1 [file ijms-26-10353-s001.zip › SupplementaryMaterialsLegends.pdf]

Supplementary Materials: The following supporting information can be downloaded at https://www.mdpi.com/article/10.3390/ijms262110353/s1 . **Table S1**. Sampling details of 205 avocado tree samples. **Table S2**. Overall statistics of sequencing data including number of reads and total number of bases (bp). **Table S3**. Statistics of sequencing data after quality control including high-quality reads count (HQ reads), percentage of high-quality reads relative to the total number of original reads (HQ reads%), number of bases in high-quality reads (HQ bases bp), and percentage of bases in high-quality reads relative to the total number of original bases (HQ bases%). **Table S4**. Statistics of mapping rate including total number of reads (total reads), number of reads aligned to the reference genome including single-end and paired-end alignments (mapped reads), and percentage of reads aligned to the reference genome relative to the total number of reads (mapping rate). **Table S5**. Statistics of coverage at 1×, 4×, 10× and 20× mean minimum depth per sample. **Table S6**. Statistics of SNPs per sample including number of homozygous genotypes consistent with the reference genome (HOM_REF), number of heterozygous genotypes (HET), number of homozygous genotypes inconsistent with the reference genome (HOM_ALT), number of transitions (Ts, substitutions between purines and purines, or pyrimidines and pyrimidines), number of transversions (Tv, substitutions between purines and pyrimidines), and ratio of transitions to transversions (Ts/Tv). **Table S7**. Statistics of SNP annotation into markers within exonic regions, synonymous and nonsynonymous substitutions, variants causing a gain (stopgain) or a loss (stoploss) of a stop codon, SNPs within 2 bp of a splicing junction (splicing), markers within intronic and intergenic regions, as well as those 1 kb upstream of a transcription start site (upstream), 1 kb downstream of a transcription termination site (downstream), or both. **Table S8**. Main three principal components from an unsupervised PCA genetic clustering in 205 avocado tree samples with 64,310,961 lcWGS-derived SNP markers. **Table S9**. Q matrix from an admixture-based unsupervised genetic clustering (K = 5) in 205 avocado tree samples with all lcWGS-derived SNP markers. **Table S10**. Three principal components from a PCA unsupervised genetic clustering in 205 avocado tree samples with 9,826 lcWGS-derived SNP markers. **Table S11**. Main two principal components from an unsupervised genetic PCA clustering in 68 racially pure (>80% ancestry to a single genetic cluster) avocado tree samples with 9,826 high-quality SNP markers. **Table S12**. Subset of 254 SNPs significantly associated with racial classification (K = 5) in 68 racially pure (>80% ancestry to a single genetic cluster) avocado tree samples as determined through linear mixed models (LMMs), F statistic, p-value and R^2 are shown for each associated marker. **Table S13**. Main two principal components from an unsupervised PCA genetic clustering in 68 racially pure (>80% ancestry to a single genetic cluster) avocado tree samples with 254 SNPs associated with racial classification (K = 5) as determined through LMMs. **Figure S1**. Sequencing depth and cumulative sequencing depth. **Figure S2**. Cross-validation (CV) error from the admixture-based unsupervised genetic clustering (from K = 2 to K = 10) in 205 avocado tree samples with 64,310,961 lcWGS-derived SNP markers. **Figure S3**. Admixture-based unsupervised genetic clustering (from K = 2 to K = 10) in 205 avocado tree samples with 64,310,961 lcWGS-derived SNP markers. **Figure S4**. Principal component analysis (PCA) unsupervised genetic clustering with 9,826 high-

quality SNP markers in all 205 avocado tree samples, so that racial controls are labeled and colored top down from left to right, as follows: pink for Mexican (ME) race, light blue for Guatemalan (GU) race, light green for West Indian (WI) race, dark blue for Caribbean Colombian (CoCA), dark green for Andean Colombian (CoA), and light gray for avocado samples identified as hybrids between different genetic groups. **Figure S5**. Bayesian phylogenetic inference (*i.e.*, GTR+Γ substitution model, with four chains, a chain temperature of 0.8, and 10×10^6 generations, the latter sampled every 1000 after discarding the first 25% as burn-in) in 68 racially pure (>80% ancestry to a single genetic cluster) avocado tree samples using 254 SNPs associated with racial classification ($K = 5$) as determined through LMMs. **Figure S6**. Flowchart summarizing the overall analysis pipeline (blue boxes refer to input data, green boxes indicate analytical tools, and orange boxes mark obtained/prospective results).
