# Supplementary figures and images for: Whole Genome Resequencing of 205 Avocado Trees Unveils the Genomic Patterns of Racial Divergence in the Americas
Source: Int J Mol Sci. 2025 Oct 24;26(21):10353. doi: 10.3390/ijms262110353 (PMC12610134; doi:10.3390/ijms262110353)

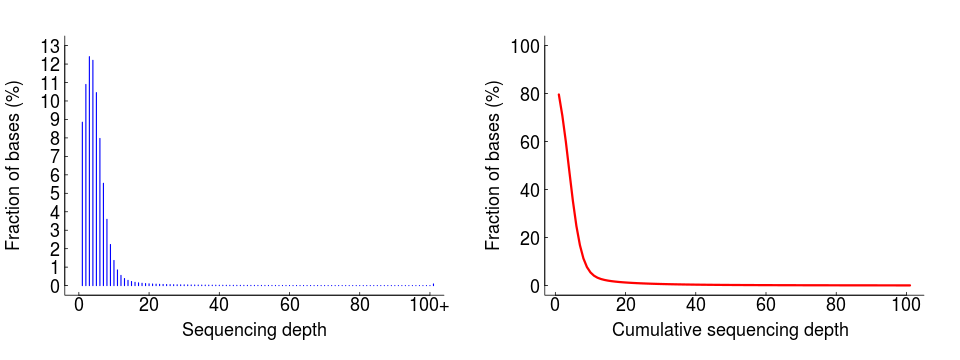

Supplement: Supplementary file 1 [file ijms-26-10353-s001.zip › FigureS1.png]

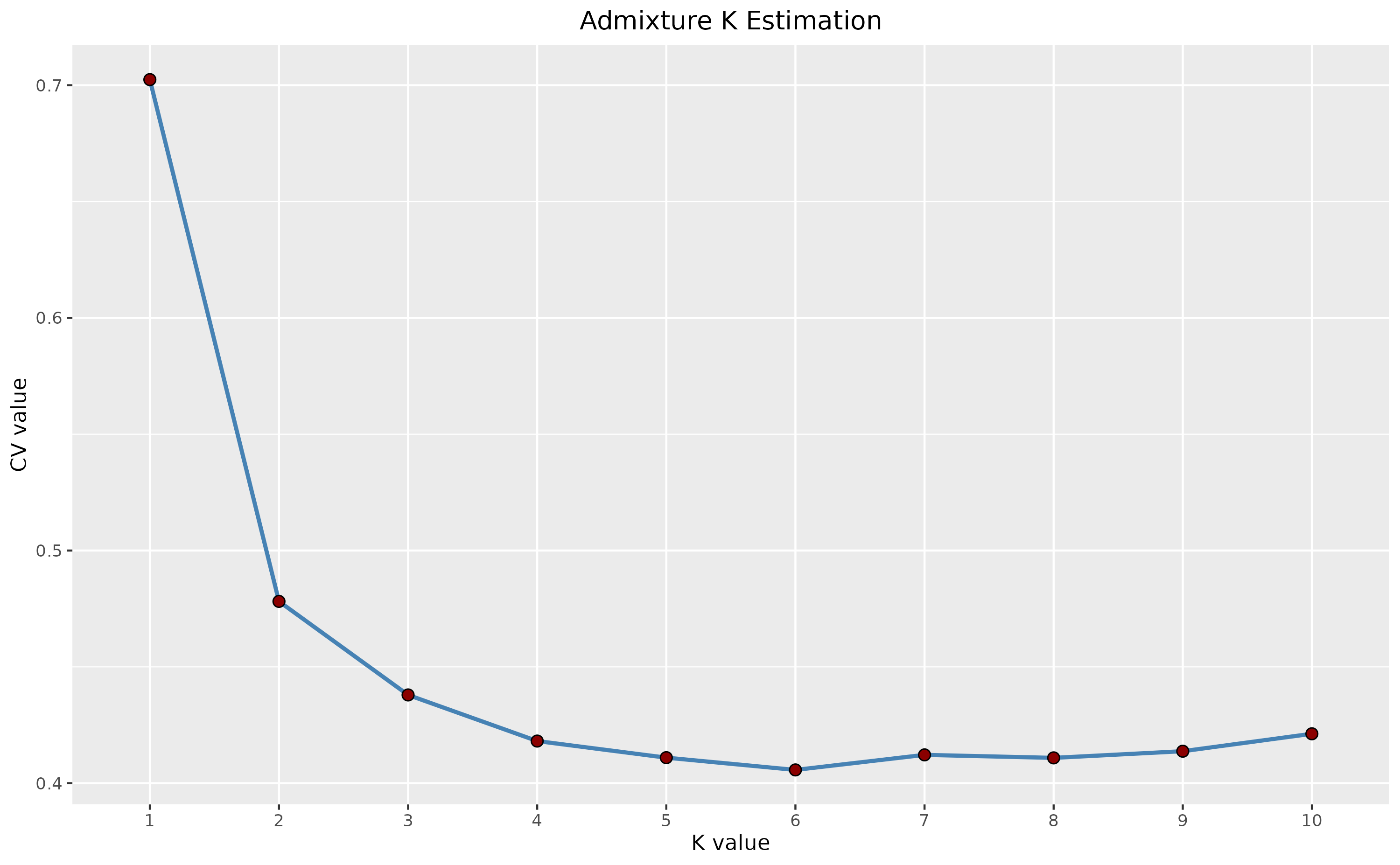

Supplement: Supplementary file 1 [file ijms-26-10353-s001.zip › FigureS2.png]

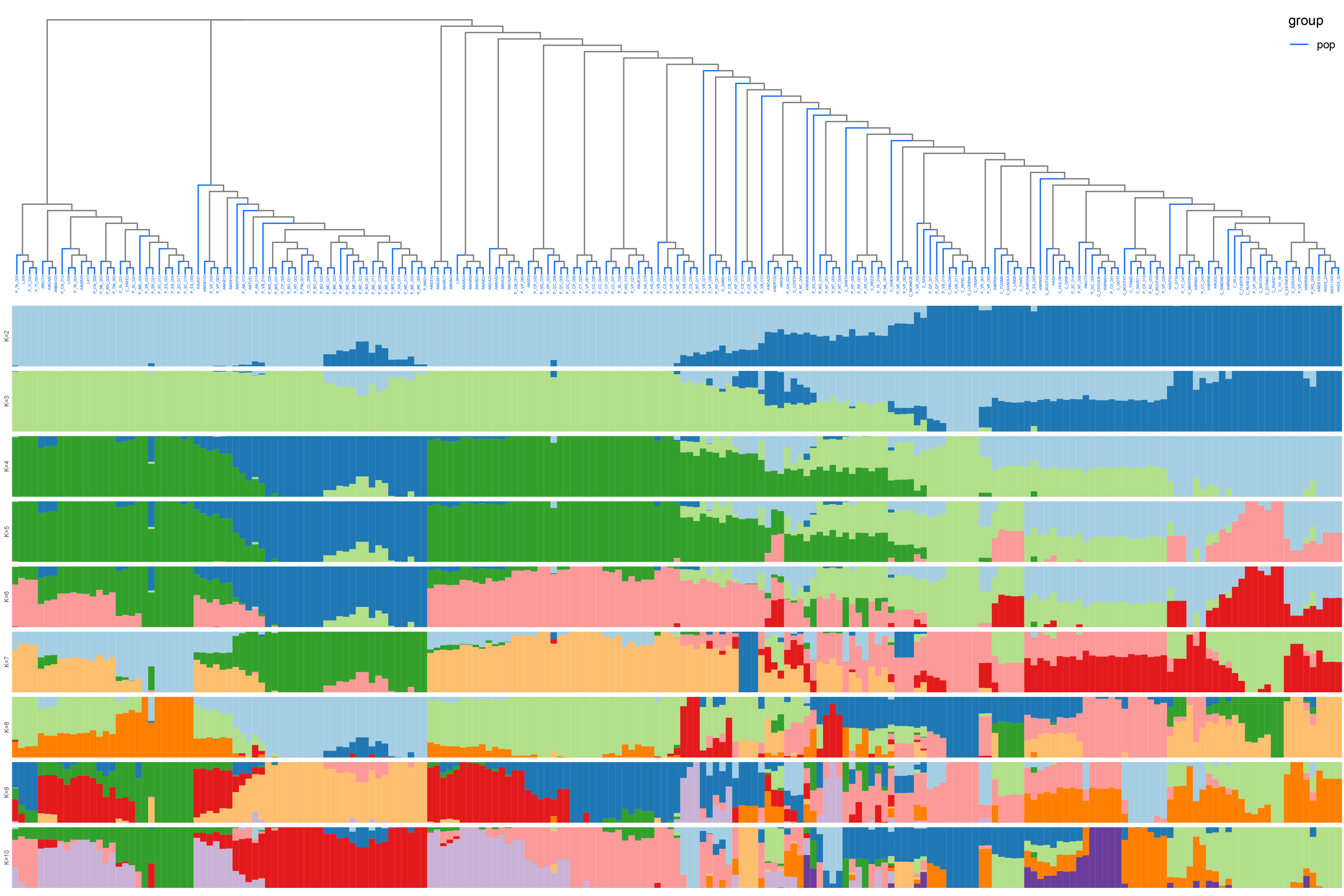

Supplement: Supplementary file 1 [file ijms-26-10353-s001.zip › FigureS3.png]

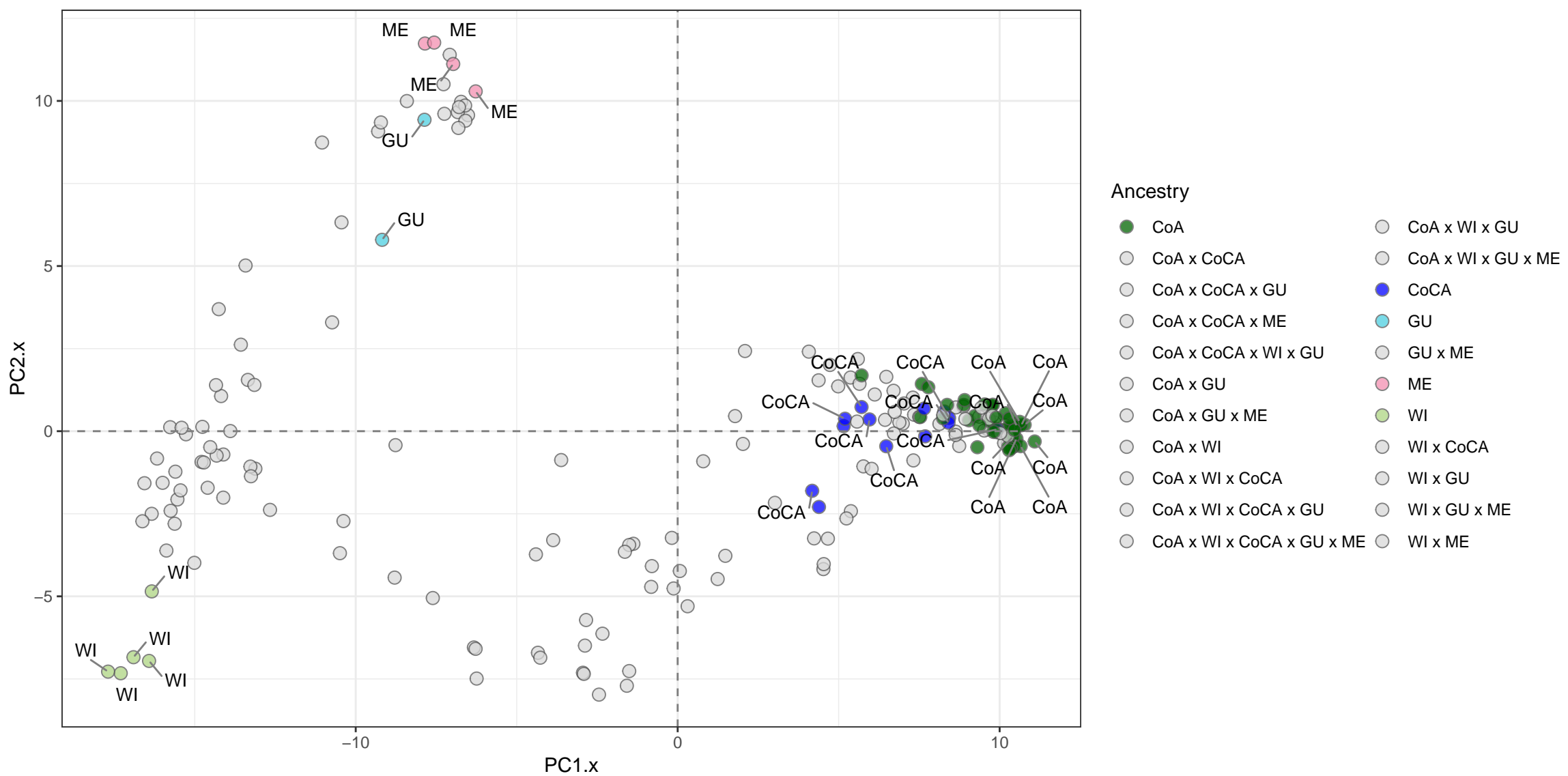

Supplement: Supplementary file 1 [file ijms-26-10353-s001.zip › FigureS4.pdf]

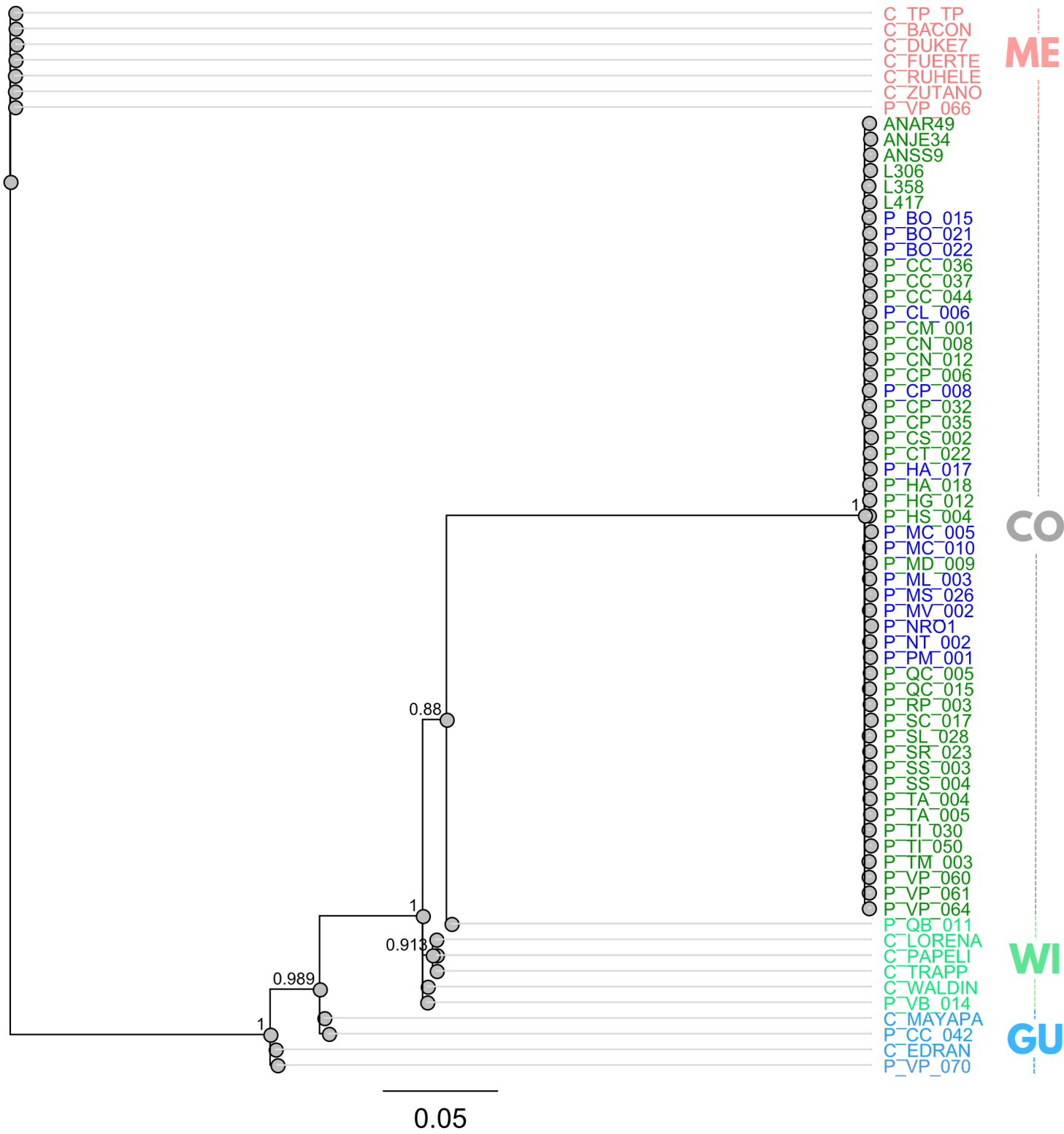

Supplement: Supplementary file 1 [file ijms-26-10353-s001.zip › FigureS5.pdf]

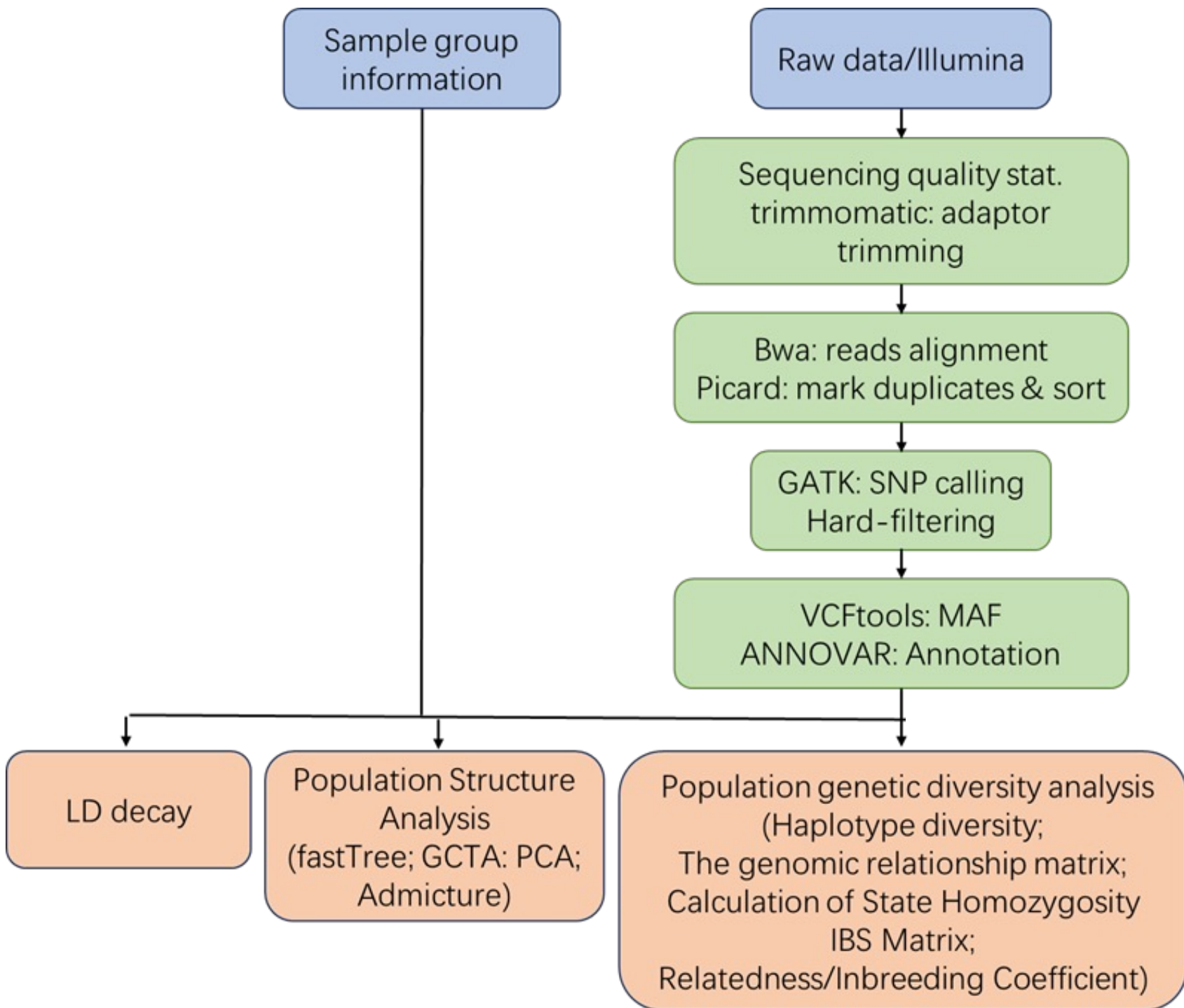

Supplement: Supplementary file 1 [file ijms-26-10353-s001.zip › FigureS6.pdf]
